# Андрогены и болезнь Паркинсона: роль у человека и в эксперименте

**DOI:** 10.14341/probl13148

**Published:** 2022-12-20

**Authors:** А. У. Хамадьянова, К. О. Кузнецов, Э. И. Гайфуллина, Д. А. Каландин, Р. Р. Хамидуллина, И. Ф. Халитова, Р. М. Фаизов, Н. О. Камалетдинова, Б. Ф. Асланова, А. Г. Накиева, Л. Э. Бурангулова, Г. О. Гайсина

**Affiliations:** Башкирский государственный медицинский университет; Башкирский государственный медицинский университет; Башкирский государственный медицинский университет; Первый Санкт-Петербургский государственный медицинский университет им. акад. И.П. Павлова; Башкирский государственный медицинский университет; Башкирский государственный медицинский университет; Башкирский государственный медицинский университет; Башкирский государственный медицинский университет; Ростовский государственный медицинский университет; Башкирский государственный медицинский университет; Башкирский государственный медицинский университет; Башкирский государственный медицинский университет

**Keywords:** болезнь Паркинсона, тестостерон, андрогены, дигидротестостерон, половые различия, эстрогены

## Abstract

Болезнь Паркинсона (БП) является вторым по распространенности нейродегенеративным заболеванием после болезни Альцгеймера. Имеются данные, что БП имеет более широкую распространенность среди мужчин, что свидетельствует об имеющейся роли половых гормонов в патогенезе развития заболевания. В статье представлен обзор исследований, посвященных изучению половых различий в заболеваемости и симптомах БП. Медикаментозная терапия андрогенами, предшественниками андрогенов, антиандрогенами и препаратами, модифицирующими метаболизм андрогенов, доступна для лечения различных эндокринных состояний, имея трансляционное значение для БП, но ни один из этих препаратов еще не показал достаточной эффективности. Хотя в настоящее время доказано, что БП более распространена у мужчин, чем у женщин, андрогены не всегда оказывают какое-либо влияние на симптомы или прогрессирование заболевания. Ингибиторы 5α-редуктазы показали нейропротекторную и антидискинетическую активность и нуждаются в дальнейшем исследовании. Несмотря на то что нейропротекторный эффект дутастерида наблюдался только до повреждения дофаминовых нейронов, отсутствие негативного влияния делает его привлекательным препаратом для применения у пациентов с БП благодаря его антидискинетическим свойствам.

## Abstract (en)

Parkinson’s disease (PD) is the second most common neurodegenerative disease after Alzheimer’s disease. There is evidence that PD has a wider prevalence among men, which indicates the existing role of sex hormones in the pathogenesis of the disease. The article presents an overview of studies devoted to the study of sex differences in the incidence and symptoms of PD. Drug therapy with androgens, androgen precursors, antiandrogens and drugs that modify androgen metabolism is available for the treatment of various endocrine conditions, having translational significance for PD, but none of these drugs has yet shown sufficient effectiveness. Although PD has now been proven to be more common in men than in women, androgens do not always have any effect on the symptoms or progression of the disease. 5α-reductase inhibitors have shown neuroprotective and anti-dyskinetic activity and need further investigation. Despite the fact that the neuroprotective effect of dutasteride was observed only before damage to DA neurons, the absence of a negative effect makes it an attractive drug for use in patients with PD due to its anti-dyskinetic properties.

## ВВЕДЕНИЕ

Болезнь Паркинсона (БП) является хроническим медленно прогрессирующим нейродегенеративным неврологическим заболеванием, в патогенезе которого лежат обширное разрушение и гибель дофаминовых нейронов в черной субстанции, а такжев других отделах центральной нервной системы [1]. В Европе средняя распространенность БП составляет 108–257 случаев на 100 000 населения. В старшей возрастной группе (старше 65 лет) распространенность увеличивается в 6–10 раз и составляет 1289–1500 на 100 000 населения. Самые низкие показатели распространенности отмечаются в Ливии — 31,4, Эфиопии — 7, Танзании — 20, Нигерии — 58–67 на 100 000 населения, что объясняется меньшей генетической предрасположенностью негроидной расы [2]. Клиническими признаками БП являются брадикинезии, тремор покоя и постуральная неустойчивость [1]. Немоторные симптомы включают различные когнитивные, нервно-психические, вегетативные исенсорные нарушения [3].

Возникновение БП может быть связано с генными мутациями, однако этиология большинства случаев БП в настоящее время неизвестна и, скорее всего, включает совокупность генетических, эпигенетических и экологических факторов риска [4].

Половые различия при БП были зарегистрированы в эпидемиологических и клинических исследованиях [5]. Эпидемиологические исследования зафиксировали, что как заболеваемость, так и распространенность БП выше у мужчин, чем у женщин, мужчины как минимум в 1,5 раза более склонны к развитию БП [6–13]. У мужчин манифестация БП происходит в среднем на 2 года раньше [14][15]. Репродуктивное здоровье женщин имеет корреляцию с возрастом начала БП [14][16][17], предполагая, что эстрогены воздействуют как протективный агент. Эпидемиологические данные о половых различиях при БП свидетельствуют о возможной полезной активности женских половых гормонов, и этот аспект был широко рассмотрен в литературе [5][18][19]. Однако андрогенам уделяется меньше внимания в отношении их потенциального воздействия на БП. Целью настоящего исследования является анализ литературы, посвященной оценке влияния андрогенов на БП у человека и в эксперименте.

## Эндогенные андрогены и БП

Тестостерон является одним из основных андрогенных стероидов, синтезируемых яичком. Тестостерон биотрансформируется в дигидротестостерон 5α-редуктазой или в эстрадиол в процессе ароматизации [20]. Действие андрогенов опосредовано связыванием с классическим или мембранным андрогенным рецептором (АР) [21][22]. Вариабельность АР, которая имеется у человека, проявляется различием в их функционировании при взаимодействии с андрогенами [23][24].

В процессе старения уровень тестостерона у мужчин постепенно снижается, начиная с четвертого или пятого десятилетия [25]. Частота андрогенного дефицита увеличивается до 12, 19, 28 и 49 для мужчин старше 50, 60, 70 и 80 лет соответственно [26]. Поскольку заболеваемость БП выше у мужчин, чем у женщин, важным вопросом является определение того, существует ли связь между уровнями андрогенов и развитием БП у мужчин (табл. 1). По результатам двух исследований, проведенных на небольшом количестве пациентов с БП, были обнаружены более низкие уровни тестостерона [27][28], чем в процессе нормального старения [26], однако эти данные должны быть подтверждены в более масштабных исследованиях. Количество АР также может играть важную роль в эффекте андрогенов при БП. Тем не менее уровни мРНК АР в черной субстанции не имели значимых различий между пациентами с БП мужского и женского пола и соответствовали значениям, полученным в контрольной группе [29]. Поскольку тестостерон способен синтезироваться в головном мозге, его уровни в плазме не всегда отражают объективную картину при БП. Насколько нам известно, измерения уровня тестостерона в головном мозге пациентов с БП не проводились.

**Table:** Таблица 1. Клинические исследования, посвященные оценке уровня тестостерона при БПTable 1. Clinical studies evaluating testosterone levels in Parkinson's disease Примечание: БП — болезнь Паркинсона; UPDRS (Unified Parkinson’s Disease Rating Scale) — унифицированная шкала оценки болезни Паркинсона; Часть I — оценка мышления, поведения и настроения; Часть II — самооценка повседневной жизнедеятельности; Часть III — оценка моторной функции; Часть IV — флуктуации; АР — андрогенный рецептор.

Эндогенные андрогены и БП
Исследование	Выборка	Основные результаты
БП и уровень тестостерона
M.S. Okun и соавт. [27]	68 пациентов с БП	Распространенность низкого уровня тестостерона у больных БП составила 35%
M.S. Okun и соавт. [28]	50 из 91 пациента с БП, прошедших скрининг на уровень свободного тестостерона	Половина пациентов с БП, которые были обследованы, имели низкий уровень тестостерона
Заболеваемость БП при снижении уровня тестостерона
S.D. Chung и соавт. [31]	1335 пациентов с раком предстательной железы сравнивались с 4005 пациентами такого же возраста	Андрогенная депривационная терапия у пациентов с раком предстательной железы не была связана с более высоким риском БП
J.W. Young и соавт. [32]	38 931 пациент с раком предстательной железы на непрерывной андрогенной депривационной терапии и 34 272 сопоставимых пациента	Андрогенная депривационная терапия у пациентов с раком предстательной железы была связана с более низким риском БП

Терапия симптомов БП андрогенами и антиандрогенами
Исследование	Выборка	Схема терапии	Основные результаты
Тестостерон
M.S. Okun и соавт. [46]	Проспективное исследование с участием 10 мужчин с дефицитом тестостерона и БП	Однократная суточная доза местного геля тестостерона (5 г/сут Андрогеля (эквивалент 5 мг/сут тестостерона)) в течение 1 мес. Шесть из 10 пациентов также наблюдались в течение 3 мес	Нет влияния на части II и III UPDRS и шкалу оценки дискинезий Обесо. Часть IV UPDRS улучшилась через 1 мес, но не через 3 мес. Часть I UPDRS улучшилась в процессе 3-месячного наблюдения
E. Mitchell и соавт. [47]	Описание клинического случая пациента 80 лет с БП и дефицитом тестостерона	Внутримышечное введение 100 мг смеси эфиров тестостерона ежемесячно на протяжении 3 мес, с последующим увеличением дозы до 250 мг ежемесячно	Уменьшение тремора в состоянии покоя и улучшение мелкой моторики после введения тестостерона коррелировало с уровнем тестостерона в сыворотке крови
M.S. Okun и соавт. [45]	Двойное слепое плацебо-контролируемое исследование (15 пациентов с БП в группе плацебо, 15 пациентов с БП в группе тестостерона)	200 мг/мл тестостерона энантата каждые 2 нед в течение 8 нед	Влияния на шкалу UPDRS не выявлено
M.S. Okun и соавт. [27]	Ретроспективный анализ пяти пациентов с сочетанием БП и дефицита тестостерона	Однократная суточная доза местного геля тестостерона (5 г/сут андрогена (эквивалент 5 мг/сут тестостерона)).	Несколько пациентов с БП отметили улучшение симптомов БП, но это не всегда было связано с изменением моторной оценки UPDRS
Ингибиторы 5α-редуктазы
M. Bortolato и соавт. [70]	Описание клинического случая двух пациентов с БП, которые имели патологическую страсть к азартным играм	Финастерид 5 мг/сут	Финастерид ослаблял патологическую страсть к азартным играм у пациентов с БП
Ингибиторы АР
H.A. Teive и соавт. [59]	Описание клинического случая 72-летнего мужчины с БП и застойной сердечной недостаточностью	Спиронолактон 100 мг/сут	Ухудшение части III UPRDS. После отмены спиронолактона двигательная функция возвращается к исходным значениям

Другим важным аспектом, который потенциально может оказывать влияние на уровень тестостерона, является дофаминергическая терапия. Предшественник дофамина (ДА) леводопа (золотой стандарт лечения БП) или агонист рецепторов ДА прамипексол не снижают уровень тестостерона, а, как сообщается, немного увеличивают его, поэтому снижение уровня тестостерона, по-видимому, не связано с дофаминергическими препаратами [30].

Кроме того, использование андрогенной депривационной терапии у пациентов с раком предстательной железы не имело корреляции с повышением риска развития БП [31][32] (табл. 1), предполагая, что низкий уровень андрогенов не является фактором риска развития БП. Риск развития паркинсонизма по результатам исследования был ниже у пациентов, проходящих андрогенную депривационную терапию, что свидетельствует о ее возможном нейропротекторном эффекте [32]. Таким образом, андрогены не оказывают протекторной функции, однако могут усиливать токсическое действие ДА.

## Эндогенные андрогены и животные модели БП

S. Khasnavis и соавт. изучали влияние кастрации на риск развития БП у мышей [33]. Кастрация у молодых самцов увеличивала глиальную активацию, снижала стриарные уровни ДА и количество тирозингидроксилаза-положительных нейронов в полосатом теле и черной субстанции, а также вызывала нарушение двигательной активности, однако у взрослых самцов кастрация не вызывала ни одного из вышеперечисленных эффектов [33]. При кастрации крыс, паркинсонизм у которых моделировался путем повреждения дофаминергических нейронов черной субстанции мозга при помощи введения в ее компактную часть селективного нейротоксина 6-гидроксидофамина (6-OHDA), было отмечено снижение токсического действия повреждающего агента (табл. 2) [34–36].

**Table:** Таблица 2. Влияние андрогенных и антиандрогенных соединений на животные модели БПTable 2. Effects of Androgenic and Antiandrogenic Compounds on Animal Models of Parkinson's Disease Примечание: ДГЭА — дегидроэпиандростерон; МФТП — 1-метил-4-фенил-1,2,3,- тетрагидропиридин; 6-OHDA — 6-гидроксидофамин.

Животные модели: эффект от кастрации	Основные результаты
6-OHDA-индуцированная модель БП крыс	После кастрации у самцов крыс отмечалось снижение токсического действия 6-OHDA [34][35]
МФТП-индуцированная модель БП мышей	Снижения токсичности МФТП после кастрации самцов мышей не наблюдалось [37][38]
Животные модели: эффект от лечения андрогенами и антиандрогенами	Обнаружен положительный эффект	Эффект отсутствует
Оценка нейропротекции
Самцы МФТП мышей	Дутастерид [39, 40]	Тестостерон [50]
ДГЭА	Дигидротестостерон [50]
-	Финастерид [39]
Кастрированные самцы МФТП мышей	-	Тестостерон [38]
Гонадэктомизированные самки и самцы 6-OHDA крыс	-	Дигидротестостерон [34][51]
Оценка влияния на дискинезии
Самки и самцы 6-OHDA-крыс	Финастерид [71]	-
Самцы 6-OHDA-крыс	Финастерид [72]	-
Самцы 6-OHDA-крыс	Дутастерид [72]	-

E. Antzoulatos и соавт. сообщили, что содержание стриарного ДА и его метаболита дигидрофенилуксусной кислоты у кастрированных пожилых самцов мышей (6 мес) не имело достоверных различий с интактными мышами и снижалось при моделировании БП путем введения нейротоксина 1-метил-4-фенил-1,2,3,5-тетрагидропиридина (МФТП) [37].

При иммуногистохимическом исследовании стриарной тирозингидроксилазы у молодых (10–12 нед) самцов мышей не было выявлено значимых различий между кастрированными и интактными животными, а при введении МФТП наблюдался дефицит тирозингидроксилазы [38]. Вышеуказанные исследования имели различия в виде и возрасте лабораторных животных, а также в способе моделирования БП. Кроме того, у кастрированных самцов 6-OHDA-крыс и самцов мышей, получавших МФТП, терапия тестостероном или дигидротестостероном не оказывала влияния на вызванное токсинами поражение [34][38].

Таким образом, кастрация на животных моделях не увеличивала восприимчивость к токсинам, повреждающим нигростриарную систему, а лечение тестостероном или дигидротестостероном не оказывало никакого эффекта.

Сообщалось, что у мышей, получавших МФТП, были снижены уровни плазменного и мозгового тестостерона и дигидротестостерона по сравнению с контрольной группой [39][40]. Клетки Лейдига являются основным местом синтеза эндогенного тестостерона в физиологических условиях, у МФТП-мышей было зарегистрировано снижение количества клеток Лейдига [41], что объясняет снижение уровня тестостерона и дигидротестостерона в данной модели БП. На активность ферментов стероидогенеза также может оказывать влияние воздействие активных форм кислорода [42][43], которые образуются после введения МФТП. В 6-OHDA-индуцированной модели БП крыс при оценке уровней тестостерона и дигидротестостерона в коре головного мозга по сравнению с контролем не было обнаружено достоверных различий, однако наблюдались различия в метаболизме прогестерона [44].

## Терапия андрогенами при БП

Клинические исследования, посвященные оценке влияния заместительной терапии тестостероном на моторные проявления БП, на сегодняшний день весьма ограничены и характеризуются малой выборкой, а также отсутствием последовательности в результатах (табл. 1).

M.S. Okun и соавт. проводили двойное слепое плацебо-контролируемое исследование, в котором в течение 8 нед оценивали влияние тестостерона энантата на моторные нарушения у пациентов с БП, имеющих уровень тестостерона в пределах нижней границы нормы [45]. Оценка двигательной функции проводилась с использованием шкалы UPDRS (Unified Parkinson’s Disease Rating Scale). По результатам исследования авторы не выявили какого-либо положительного влияния терапии тестостероном у пациентов с БП [45]. В проспективном исследовании с участием 10 мужчин с БП, у которых наблюдался дефицит тестостерона, баллы UPRDS IV (флуктуации) улучшались через 1 мес, однако устойчивого улучшения через 3 мес отмечено не было [46]. В этом же исследовании терапия тестостероном не показала значимого влияния на баллы UPRDS II (повседневная активность) и III (моторная функция), а также не было изменений по шкале оценки дискинезий Обесо [46]. E. Mitchell и соавт. описали клинический случай 80-летнего мужчины с БП и дефицитом тестостерона, у которого на фоне введения тестостерона наблюдались значительное снижение тремора в состоянии покоя, а также улучшение мелкой моторики.

В ретроспективном анализе пяти пациентов с БП, получавших тестостерон в целях восполнения его дефицита, несколько пациентов отметили улучшение симптомов БП, однако оно не отражалось в улучшении по шкале UPRDS [27]. Как отмечают авторы, положительная динамика может быть результатом влияния тестостерона на настроение и энергию, а не прямого влияния на симптомы БП.

Следует учитывать, что три из четырех исследований, представленных в этом разделе, включали пациентов с БП, имеющих пониженный уровень тестостерона. Неспецифические признаки, связанные с дефицитом тестостерона, включают снижение энергии, нарушение физической работоспособности и ограничение подвижности [48][49]. Терапия тестостероном у пожилых мужчин с его дефицитом оказывала положительный эффект на вышеуказанные проявления [48][49]. Таким образом, поскольку терапия тестостероном может непосредственно воздействовать на симптомы, связанные с дефицитом тестостерона, неясно, улучшение происходит от нормализации уровня тестостерона или от прямого воздействия на двигательные проявления БП. Положительное влияние при БП может также объясняться превращением тестостерона в 17β-эстрадиол ароматазой.

## Терапия андрогенами на животных моделях БП

У самцов мышей терапия тестостероном не вызывала какого-либо протективного эффекта в МФТП-индуцированной модели БП [38][50] (табл. 2). Отсутствие эффекта тестостерона может быть результатом недостаточного превращения в эстрадиол или отсутствия положительного эффекта от стимуляции АР. Чтобы конкретно исследовать роль стимуляции АР в нейропротекции, дигидротестостерон (наиболее мощный андроген) является более подходящим соединением, чем тестостерон, поскольку он не ароматизирован эстрадиолом. Исследования, проведенные на МФТП-самцах мышей и гонадэктомированных 6-OHDA-самках и самцах крыс, не обнаружили положительного влияния от лечения дигидротестостероном [34][50][51], предполагая, что стимуляция АР неэффективна в индукции нейропротекции. Учитывая отсутствие эффектов как тестостерона, так и дигидротестостерона, эти результаты свидетельствуют о том, что тестостерон в головном мозге нетрансформируется в эстрадиол в достаточной концентрации для достижения нейропротекции.

При введении тестостерона пожилым крысам наблюдаются улучшение двигательного дефицита, а также увеличение транспортеров ДА и тирозингидроксилазы в полосатом теле и черной субстанции [52][53]. Тем не менее такие результаты получены в процессе нормального старения, а не при патологических состояниях. В условиях, когда присутствует окислительный стресс, например, у взрослых самцов крыс, получавших резерпин, тестостерон ухудшал дефицит в поведении и внигростриарной дофаминергической системе [52].

Дигидротестостерон может метаболизироваться в 3β-диол, который является агонистом рецепторов эстрогена [54]. Ранее было описано снижение уровней тестостерона, дигидротестостерона и 3β-диола у самцов МФТП мышей [40][55]. У мужчин с БП сообщалось о снижении уровня 17β-эстрадиола и тестостерона [56]. Снижение гонадных андрогенов у мужчин с БП и у МФТП-мышей связано с нарушением активности клеток Лейдига. Следовательно, роль 3β-диола при БП определить сложно, поскольку он является более слабым агонистом рецепторов эстрогена (сродство связывания к ERα 6 нМ (против 0,13 для 17β-эстрадиола) и 2 нМ к ERβ (против 0,12 для 17β-эстрадиола)) [54], чем 17β-эстрадиол, и его уровни снижаются в зависимостиот метаболического предшественника — дигидротестостерона.

Таким образом, клинические исследования и исследования на животных не подтверждают то, что андрогены могут изменять риск развития БП. Потенциальное благотворное влияние тестостерона в сочетании с антипаркинсоническими препаратами при БП требует проведения более масштабных исследований, чтобы сделать однозначный вывод.

## Антиандрогенная терапия при БП

Антиандрогенная терапия включает применение препаратов, ингибирующих гипоталамо-гипофизарно-гонадную ось, включая модуляторы гонадотропин-ингибирующего гормона (лейпролид, гозерелин и трипторелин), антагонисты гонадолиберина (дегареликс), ингибиторы АР (ципротерон, спиронолактон, эплеренон и флутамид) и ингибиторы 5α-редуктазы (финастерид и дутастерид). Ингибиторы АР и 5α-редуктазы оказывают антиандрогенное действие, и некоторые их них были исследованы напациентах с БП. Основными представителями ингибиторов АР являются спиронолактон и эплеренон, которые также оказывают влияние на минералокортикоидные рецепторы [57][58].

H.A. Teive и соавт. описали клинический случай, в котором применение спиронолактона усугубляло симптомы БП [59]. В исследовании на крысах низкие дозы флутамида уменьшали каталепсию, индуцированную галоперидолом, а более высокие дозы усиливали ее выраженность [60]. В дофаминергической клеточной линии (клетки N27) флутамид ингибировал тестостерон-индуцированный апоптоз [61], который был опосредован мембранными АР в клетках N27 [62]. P. Haussermann и соавт. описали клинический случай, в котором лечение низкими дозами ципротерона ацетата уменьшало гиперсексуальность у мужчин с БП и деменцией без соответствующих побочных эффектов [63]. Исследований, посвященных оценке действия ингибиторов АР при БП, очень мало, а их результаты не являются однозначными.

5α-редуктаза

5α-редуктаза является ферментом, который катализирует превращение прогестерона в дигидропрогестерон, а также метаболизирует тестостерон в дигидротестостерон. Фермент имеет две изоформы, которые экспрессируются в головном мозге [64]. В мозге крыс изоформа 5α-редуктазы 2 локализуется в нейронах, тогда как изоформа 1 экспрессируется в глиальных клетках [65][66], что говорит о различных функциях этих изоформ в регуляции нейроэндокринных процессов. Ингибиторы 5α-редуктазы, такие как финастерид и дутастерид, используются в клинической практике для лечения доброкачественной гиперплазии предстательной железы и андрогенной алопеции [67]. Финастерид избирательно ингибирует 5α-редуктазу 2 типа, тогда как дутастерид обладает более высокой эффективностью за счет ингибирующего действия на обе изоформы 5α-редуктазы [68].

Ингибиторы 5α-редуктазы

Исследования показали роль ингибиторов 5α-редуктазы в дофаминергической нейротрансмиссии, с потенциальными терапевтическими эффектами при нескольких расстройствах, связанных с дофаминергической гиперактивностью [69]. Что касается БП, исследование, проведенное на двух пациентах с патологической склонностью к азартным играм, вызванной дофаминергическими препаратами, показало, что лечение финастеридом уменьшило выраженность данного осложнения [70] (табл. 1).

Как у самок, так и у самцов 6-OHDA-крыс финастерид снижает развитие леводопа-индуцированных дискинезий [71]; такой же эффект наблюдается при применении дутастерида у самцов 6-OHDA-крыс [72]. Для получения этого эффекта требуется более низкая доза дутастерида по сравнению с финастеридом [72]. Кроме того, дутастерид не оказывает влияния на леводопа-индуцированную двигательную активацию, в отличие от финастерида [72]. Сообщалось, что финастерид ослабляет психотические осложнения агонистов рецепторов ДА (D1 и D3), что позволяет предположить аналогичный механизм его действия на данные рецепторы при уменьшении дискинезий [73][74]. Кроме того, как дутастерид, так и финастерид предотвращают леводопа-индуцированную экспрессию дофаминового рецептора D1 и взаимодействие рецепторов D1–D3 [72]. Эти исследования показывают, что ингибиторы 5α-редуктазы могут оказывать положительный эффект для уменьшения побочных эффектов, связанных с дофаминергическими препаратами, такими как леводопа-индуцированные дискинезии и компульсивное поведение.

## Антиандрогенная терапия на животных моделях БП

Ингибиторы 5α-редуктазы: нейропротекторное действие

Поскольку ингибиторы 5α-редуктазы блокируют превращение прогестерона в дигидропрогестерон, а также тестостерона в дигидротестостерон и, таким образом, гипотетически увеличивают уровень 17β-эстрадиола путем ароматизации тестостерона, они могут оказывать нейропротекторное действие за счет повышения уровня нейропротекторных стероидов (табл. 2).

Введение дутастерида самцам мышей до введения МФТП предотвращало разрушение дофаминергических нейронов в черной субстанции, однако, когда дутастерид вводился после МФТП, протективного эффекта не наблюдалось и результаты были сопоставимы с контролем [39][40]; дутастерид не увеличивал токсичность МФТП. Эффективность финастерида в протекции дофаминергических нейронов у самцов МФТП мышей не была доказана [39]; возможно, это связано с его более коротким периодом полувыведения (2 ч) по сравнению с дутастеридом (31 ч) [68].

МФТП снижает уровень тестостерона в плазме крови и головном мозге, а введение дутастерида у МФТП мышей позволяет поддерживать его концентрацию в пределах референсных значений, в то время как уровень дигидротестостерона как у мышей контрольной группы, так и у МФТП-мышей, получающих дутастерид, был снижен [40]. Поскольку терапия тестостероном и дигидротестостероном у самцов МФТП-мышей не вызывала никакого защитного эффекта [50], кажется маловероятным, что поддержание физиологического уровня тестостерона может быть одним из механизмов, с помощью которых дутастерид предотвращал токсичность МФТП.

Хотя уровень 17β-эстрадиола не был проанализирован в полосатом теле и черной субстанции, он определялся в плазме крови и в одном полушарии головного мозга интактных самцов мышей. Его уровни становились неопределяемыми как после введения МФТП, так и на фоне лечения МФТП-мышей дутастеридом, предполагая, что протективный эффект дутастерида вряд ли опосредован повышением уровня 17β-эстрадиола [39][40].

Концентрация прогестерона в плазме крови и мозге находилась в пределах референсных значений при введении дутастерида интактным мышам, однако у МФТП-мышей, которым вводили дутастерид, наблюдались повышенные уровни прогестерона [40]. Таким образом, протективный эффект дутастерида, по-видимому, связан не только с изменением содержания прогестерона, тестостерона и их метаболитов.

Дутастерид увеличивает специфическое связывание и гликозилирование дофаминового транспортера (DAT — Dophamine transporter) у интактных самцов мышей, следовательно, увеличивая перемещение ДА через клеточную мембрану [40]. S.T. Masoud и соавт. установили, что мыши с повышенной экспрессией DAT более восприимчивы к токсичности МФТП [75], однако эти данные не подтверждаются исследованием N. Litim и соавт., которые утверждают, что повышение экспрессии DAT при терапии дутастеридом не повышает токсичность МФТП, а наоборот, способствует нейропротекции [40]. Нейропротекция при терапии дутастеридом МФТП-мышей также обусловлена уменьшением нейровоспаления, которое оценивалось авторами наосновании изменения глиального фибриллярного кислого протеина [40].

## Предшественники андрогенов (дегидроэпиандростерон и прегненолон) при терапии БП

Дегидроэпиандростерон (ДГЭА) и прегненолон являются предшественниками стероидов в синтетических путях андрогенов. Прегненолон является одобренным FDA препаратом, который в настоящее время исследуется для лечения психических расстройств с нарушением дофаминергической передачи, включая биполярные расстройства, шизофрению и интоксикацию марихуаной [76]. У 6-OHDA-крыс уровни ДГЭА в полосатом теле и коре головного мозга не изменялись, тогда как уровни прегненолона были снижены в полосатом теле, но не в коре [44]. Уровни ДГЭА у МФТП-мышей были неизмененными в плазме крови, а уровни прегненолона снижались в плазме крови и повышались в головном мозге [40].

На сегодняшний день данные о прегненолоне и ДГЭА в спинномозговой жидкости, плазме крови и/или головном мозге пациентов с БП весьма ограничены. Уровни ДГЭА и его сульфатного производного не изменялись у пациентов с БП [77]. Наживотных моделях БП (МФТП обезьяны) сообщалось о благотворном влиянии ДГЭА на двигательные функции [78, 79], а также о выраженном нейропротекторном действии [80]. Существует потенциал использования прегненолона для лечения БП илеводопа-индуцированных дискинезий. Он способен восстанавливать синаптические дефекты и нормализовать гипердофаминергическую активность и аномальное ДА-зависимое поведение у потомства крыс, подвергшихся воздействию каннабиноидов во время беременности [81]. Прегненолон стабилизирует возбудимость дофаминовых нейронов и предотвращает тетрагидроканнабинол-индуцированное повышение стриарных уровней ДА. Вышеуказанные эффекты сохранялись даже после полного выведения прегненолона, что говорит о его длительных свойствах в противодействии патологическим гипердофаминергическим состояниям [81].

## ОБСУЖДЕНИЕ

Старение является основным фактором риска развития БП, что может быть обусловлено снижением функции гонад как мужчин, так и у женщин. У женщин потеря функции яичников происходит резко после наступления менопаузы, тогда как у мужчин андропауза (снижение функции гонад) имеет длительное и постепенное течение.

Учитывая половые различия при БП, указывающие на защитную роль яичниковых стероидов, которая теряется при менопаузе, и обильные литературные данные о нейропротекторной активности эстрогенов и прогестерона на животных моделях БП, заместительная гормональная терапия (эстроген и прогестерон) кажется рациональным подходом к решению проблемы [18]. Однако побочные эффекты такой терапии привели к поиску альтернатив, например селективные модуляторы рецепторов эстрогена, ралоксифен, специфические агонисты рецепторов эстрогена (α-, β-рецепторов и мембранного рецептора эстрогена GPER1) [18]. Как было рассмотрено выше, заместительная терапия андрогенами не дала убедительных доказательств положительного или негативного влияния при БП. Кастрация на животных моделях не увеличивала восприимчивость к токсинам, повреждающим нигростриарную систему, а лечение тестостероном или дигидротестостероном не оказывало никакого эффекта.

В нескольких клинических исследованиях был обнаружен регресс симптомов БП на фоне терапии тестостероном, однако большинство из рассмотренных нами исследований включало пациентов, имеющих пониженный уровень тестостерона. Таким образом, поскольку терапия тестостероном может непосредственно воздействовать на симптомы, связанные с его дефицитом, неясно, улучшение происходит от нормализации уровня тестостерона или от прямого воздействия на двигательные проявления БП, что требует проведения дополнительных исследований.

Интересные результаты продемонстрировали исследования, посвященные терапии БП антиандрогенными препаратами. Так, применение ингибиторов 5α-редуктазы снижало частоту развития леводопа-индуцированных дискинезий, а также ослабляло выраженность психотических осложнений агонистов ДА рецепторов. Кроме того, в нескольких исследованиях, дутастерид показал выраженный нейропротекторный эффект, точный механизм которого еще предстоит выяснить.

Что касается предшественников андрогенов, прегненолон позволяет снижать выраженность и частоту леводопа-индуцированных дискинезий за счет стабилизации возбудимости дофаминовых нейронов. Данный эффект сохранялся даже после полного выведения прегненолона, что говорит о его длительных свойствах в противодействии патологическим гипердофаминергическим состояниям.

Хотя настоящий обзор в основном был сосредоточен на воздействии андрогенов у мужчин с БП, имеются данные об изменении уровня андрогенов на протяжении всей жизни женщин. Уровни андрогенов в сыворотке крови женщин снижаются в раннем репродуктивном возрасте и остаются стабильными на протяжении всей жизни, в т.ч. после наступления менопаузы [82]. Более высокий уровень андрогенов относительно эстрогена у женщин в постменопаузальном состоянии и его роль в увеличении заболеваемости БП после менопаузы еще предстоит исследовать. Тем не менее у гонадэктомированных крыс лечение дигидротестостероном не оказывало влияние на токсичность 6-OHDA, тогда как эстрадиол проявлял выраженный протективный эффект [34], на основании чего можно предположить, что повышение уровня андрогенов у женщин не оказывает повреждающего действия на дофаминергическую систему.

Многие клеточные механизмы, способствующие нарушению функции нейронов в процессе старения, также присутствуют при БП, включая митохондриальную дисфункцию, воспаление, окислительный стресс и нарушение метаболизма ДА [83–85]. При старении наблюдается снижение синтеза ДА, рецепторов ДА и DAT, а также тирозингидроксилазных нейронов [83][85–87]. Возрастное снижение активности ДА в головном мозге приводит к когнитивной и двигательной дисфункции как у мужчин, таки у женщин [88–90]. Изменения, происходящие в процессе старения, делают дофаминовые нейроны более уязвимыми к повреждениям. Действительно, токсин МФТП вызывает большую дегенерацию дофаминовых нейронов у пожилых обезьян и мышей по сравнению с молодыми особями [91][92].

У большинства людей процесс старения протекает без возникновения БП. Этиология дегенерации дофаминовых нейронов при БП до сих пор неизвестна, вероятно, она является многофакторной и включает как генетические, так и экологические факторы [84].

Несмотря на то что уменьшение количества нигростриарных дофаминовых нейронов является основной нейропатологической причиной БП, другие группы нейронов также подвергаются дегенерации, например нейроны ядер шва, норадренергические нейроны голубого пятна, а также холинергические нейроны базального ядра Мейнерта [93]. Напротив, имеются данные, что нейротрансмиссия глутамата в головном мозге увеличивается при БП [94]. Патоморфология БП также включает в себя накопление внутриклеточных α-синуклеиновых белков, называемых тельцами Леви [95]. Учитывая, что нейродегенеративные заболевания, включая БП, связаны с воспалением, противовоспалительное действие различных стероидов может оказывать определенный терапевтический эффект [96].

Вирусные инфекции могут быть потенциальным фактором риска развития БП, и этому есть подтверждающие, хотя и не совсем последовательные эпидемиологические и фундаментальные научные доказательства [97]. S. Sadasivan и соавт. продемонстрировали, что воздействие на мышей вируса гриппа A/California/04/2009 H1N1, который вызывает воспаление мозга, усугубляет их уязвимость к паркинсоническому токсину МФТП, что приводит к повышенной потере ДА нейронов [98]. Это открытие вызывает обеспокоенность о том, что люди, перенесшие вирусные инфекции, могут быть более восприимчивы к другим потенциальным триггерам БП, которые самостоятельно не способны вызвать развитие заболевания.

Поскольку было показано, что мужчины переносили коронавирусную инфекцию (COVID-19) тяжелее, чем женщины, был рассмотрен вопрос об использовании эндокринологических препаратов с целью терапии COVID-19 [99]. Недавние исследования показали, что ингибиторы 5-α-редуктазы (финастерид и дутастерид) оказывают благотворное влияние на мужчин с COVID-19 [99–102]. Учитывая возможный рост заболеваемости БП после перенесенного COVID-19 и более высокий показатель заболеваемости мужчин обоими заболеваниями, открываются широкие возможности для перепрофилирования лекарственных средств.

Производные тестостерона и связанные с ним соединения (такие как анаболические андрогенные стероиды) могут быть полезны при БП [103]. Селективные модуляторы андрогенных рецепторов (SARM — Selective androgen receptor modulator) представляют собой соединения, разработанные как тканеселктивные лиганды АР [103][104]. SARM дают альтернативу терапии андрогенами (остеопороз, рак предстательной железы и мышечные дистрофии), но в настоящее время признаны запрещенными веществами Всемирным антидопинговым агентством [104]. Флутамид, первоначально классифицированный как ингибитор АР, в настоящее время рассматривается как SARM. Активность SARM в нормальном мозге и при БП еще предстоит исследовать.

## ЗАКЛЮЧЕНИЕ

Хотя в настоящее время доказано, что БП более распространена у мужчин, чем у женщин, андрогены не всегда оказывают какое-либо влияние на прогрессирование заболевания. Клинические и экспериментальные исследования не подтверждают, что андрогены могут изменять риск развития БП. Многочисленные антиандрогенные препараты широко доступны для лечения различных эндокринных заболеваний, что дает возможность перепрофилировать их для терапии БП. Ингибиторы 5α-редуктазы показали нейропротекторную и антидискинетическую активность и нуждаются в дальнейшем исследовании. Несмотря на то что нейропротекторный эффект дутастерида наблюдался только до повреждения ДА нейронов, отсутствие негативного влияния делает его привлекательным препаратом для применения у пациентов с БП благодаря его антидискинетическим свойствам [72]. Потенциальное благотворное влияние тестостерона в сочетании с антипаркинсоническими препаратами при БП требует проведения более масштабных исследований, чтобы сделать однозначный вывод.

## ДОПОЛНИТЕЛЬНАЯ ИНФОРМАЦИЯ

Источники финансирования. Работа выполнена по инициативе авторов без привлечения финансирования.

Конфликт интересов. Авторы декларируют отсутствие явных и потенциальных конфликтов интересов, связанных с содержанием настоящей статьи.

Участие авторов. Хамадьянова А.У. — разработка концепции и дизайна исследования, получение и анализ данных, интерпретация результатов; Кузнецов К.О. — разработка концепции и дизайна исследования, получение и анализ данных, интерпретация результатов; Гайфуллина Э.И. — разработка дизайна исследования, написание статьи; Каландин Д.А. — анализ данных, написание статьи; Хамидуллина Р.Р. — интерпретация результатов, написание статьи; Халитова И.Ф. — получение и анализ данных, редактирование статьи; Фаизов Р.М. — интерпретация результатов, редактирование статьи; Камалетдинова Н.О. — анализ данных, редактирование статьи; Асланова Б.Ф. — получение данных, редактирование статьи; Накиева А.Г. — интерпретация результатов; Бурангулова Л.Э. — написание и редактирование статьи; Гайсина Г.О. — написание и редактирование статьи. Все авторы внесли равный вклад в написание статьи и одобрили ее финальную версию перед публикацией, выразили согласие нести ответственность за все аспекты работы, подразумевающую надлежащее изучение и решение вопросов, связанных с точностью или добросовестностью любой части работы.
